# Olfactory Sensory Activity Modulates Microglial-Neuronal Interactions during Dopaminergic Cell Loss in the Olfactory Bulb

**DOI:** 10.3389/fncel.2016.00178

**Published:** 2016-07-15

**Authors:** Bryce D. Grier, Leonardo Belluscio, Claire E. J. Cheetham

**Affiliations:** Developmental Neural Plasticity Section, National Institute of Neurological Disorders and Stroke, National Institutes of HealthBethesda, MD, USA

**Keywords:** olfactory, dopaminergic neuron, microglia, plasticity, naris occlusion, 2-photon

## Abstract

The mammalian olfactory bulb (OB) displays robust activity-dependent plasticity throughout life. Dopaminergic (DA) neurons in the glomerular layer (GL) of the OB are particularly plastic, with loss of sensory input rapidly reducing tyrosine hydroxylase (TH) expression and dopamine production, followed by a substantial reduction in DA neuron number. Here, we asked whether microglia participate in activity-dependent elimination of DA neurons in the mouse OB. Interestingly, we found a significant reduction in the number of both DA neurons and their synapses in the OB ipsilateral to the occluded naris (occluded OB) within just 7 days of sensory deprivation. Concomitantly, the volume of the occluded OB decreased, resulting in an increase in microglial density. Microglia in the occluded OB also adopted morphologies consistent with activation. Using *in vivo* 2-photon imaging and histological analysis we then showed that loss of olfactory input markedly altered microglial-neuronal interactions during the time that DA neurons are being eliminated: both microglial process motility and the frequency of wrapping of DA neuron somata by activated microglia increased significantly in the occluded OB. Furthermore, we found microglia in the occluded OB that had completely engulfed components of DA neurons. Together, our data provide evidence that loss of olfactory input modulates microglial-DA neuron interactions in the OB, thereby suggesting an important role for microglia in the activity-dependent elimination of DA neurons and their synapses.

## Introduction

Microglia, the resident immune cells of the central nervous system, are known to play important roles in inflammation and phagocytosis of debris under pathological conditions (Kreutzberg, 1996). However, recent evidence suggests that microglia also play active roles in normal brain function and plasticity (Nimmerjahn et al., 2005). At the cellular level, microglia can induce apoptosis in the developing brain (Frade and Barde, 1998; Marin-Teva et al., 2004) and regulate neurogenesis in the adult brain (Walton et al., 2006; Nikolakopoulou et al., 2013; Ribeiro Xavier et al., 2015). At the synaptic level, microglia can both prune synaptic terminals during development (Paolicelli et al., 2011; Schafer et al., 2012) and promote synaptogenesis in culture or *in vivo* during motor learning (Lim et al., 2013; Parkhurst et al., 2013).

There is also mounting evidence that microglial function in sensory systems is activity-dependent. First, during early visual development, less active retinogeniculate synapses are selectively pruned by microglia (Schafer et al., 2012). Second, *in vivo* imaging has shown that microglial morphology, microglial process motility, and microglial process contact with synaptic structures in visual cortex (V1) all change with the level of visual input during the critical period (Tremblay et al., 2010). Finally, cochlear ablation results in both microglial activation and increased apposition between microglia and deafferented neurons in the ventral cochlear nucleus (Fuentes-Santamaría et al., 2012).

The OB is unusual in retaining a high level of plasticity throughout life. In particular, OB DA neurons display a remarkable level of activity-dependent plasticity: loss of sensory input drastically reduces their expression of TH within 4 days (Baker et al., 1993), while 40% of DA neurons are lost within 4 weeks (Sawada et al., 2011). Given the role of microglia in activity-dependent regulation of neuronal and synaptic density in other brain regions, we investigated whether microglia are also involved in activity-dependent elimination of OB DA neurons. Seven days after blockade of olfactory sensory input, as OB DA neurons and their synapses were being lost, microglial density increased and microglia adopted more activated morphologies. Furthermore, we found clear activity-dependent changes in microglial-neuronal interactions, providing further evidence that microglia play an activity-dependent role in the healthy brain.

## Materials and methods

### Experimental animals

All animal procedures complied with National Institutes of Health guidelines and were approved by the National Institute of Neurological Disorders and Stroke Animal Care and Use Committee. Data are from 52 male mice. Mice were kept on a 12 h light/dark cycle with food and water *ad libitum*. For initial pilot experiments, to determine the optimal duration of naris occlusion, mice were postnatal day 98 (P98) on the day of perfusion. All other mice were P56 on the day of *in vivo* imaging or transcardial perfusion. Experimental mice were: TH-Cre^+∕−^;ROSA-flox- tdTomato^+∕−^(TH-tdTom), TH-Cre^+∕−^;ROSA-flox- synaptophysin-tdTomato^+∕−^(TH-syptdTom), TH-Cre^+∕−^; ROSA -flox-tdTomato^+∕−^;CX_3_CR1^+∕GFP^ (TH-tdTom; CX_3_-GFP), or TH-Cre^+∕−^; ROSA-flox-synaptophysin -tdTomato^+∕−^;CX_3_CR1^+∕GFP^ (TH-syptdTom;CX_3_-GFP). Mouse lines were bred in-house from the following lines purchased from The Jackson Laboratory: TH-Cre^+∕−^ (#008601, www.jax.org/strain/008601, RRID:IMSR_JAX:008601), ROSA-flox-tdTomato^+∕+^ (#007909 [Ai9], www.jax.org/strain/007909, RRID:IMSR_JAX:007909), ROSA-flox-synaptophysin-tdTomato^+∕+^ (#012570 [Ai34D], www.jax.org/strain/012570, RRID:IMSR_JAX:012570) and CX_3_CR1^GFP∕GFP^ (#005582, https://www.jax.org/strain/005582, RRID:IMSR_JAX:005582).

### Unilateral naris occlusion

Mice were briefly anesthetized using isoflurane (4% in O_2_). The right naris was occluded on P49 using small plugs as described (Cummings and Brunjes, 1997). Naris occlusion was confirmed by visual inspection. Immunohistochemical confirmation of decreased TH expression in the occluded OB was performed for a subset of mice. For our initial analyses (**Figures 2, 3**) we included both the OB contralateral to the occluded naris (open OB) of naris occluded mice, and separate control mice, as control groups. While the open OB provides a within-subject control, we also included age-matched control mice in order to confirm that the changes we report are specific to loss of sensory input to the occluded OB, rather than increased air flow through the unoccluded naris resulting in compensatory changes in the open OB (Coppola, 2012).

### Transcardial perfusion and immunohistochemistry

Control and naris-occluded mice were deeply anesthetized with ketamine (300 mg/kg) and xylazine (6 mg/kg) and perfused transcardially with 0.1 M PBS, then 4% PFA. OBs were post-fixed overnight (4% PFA), cryoprotected (30% sucrose), embedded (10% gelatin), fixed/cryopreserved (15% sucrose/2% PFA) overnight and sectioned coronally at 40 μm. The cutting angle was carefully adjusted to ensure that sections contained equivalent coronal planes through the left and right OBs. For immunohistochemistry, sections were blocked for 1 h (5% horse serum/0.5% Triton X-100), followed by 48 h with primary antibody (4°C) and 2 h with secondary antibody (room temperature). Antibodies were rabbit anti-TH (Novus Biologicals, 1:2500, Novus Cat# NB 300-109 RRID:AB_350437), donkey anti-rabbit-488 (Jackson ImmunoResearch Labs Cat# 711-545-152 RRID:AB_2313584) and donkey anti-rabbit-647 (Jackson ImmunoResearch Labs Cat# 711-605-152, RRID:AB_2492288; both Jackson-ImmunoResearch, 1:500).

### Widefield and confocal imaging

Images were acquired using a Leica TCS SP5 microscope. GFP fluorescence was collected at 500–550 nm and tdTomato fluorescence at 600–650 nm. Widefield images were acquired using an HCX PLAN FLUOTAR 10x/0.30 NA air objective (0.85 μm pixel diameter). Confocal images were acquired using an HCX PL APO 40x/1.25 NA oil-immersion objective (1 Airy Unit pinhole, 0.21 μm pixel diameter).

### Determination of a time point at which to assess microglial involvement in DA neuron loss

Pilot experiments were conducted to estimate the time course of DA neuron loss following unilateral naris occlusion. We found a progressive decrease in DA neuron density following naris occlusion: 22.3% after 7 days (*n* = 2), 30.2% after 14 days (*n* = 1), and 38.4% after 21 days (*n* = 2). Based on these findings, 7 days was selected as an optimal time point at which to assess the involvement of microglia in DA neuron loss, as DA neuron loss was pronounced but still ongoing.

### Thinned-skull surgery and 2-photon imaging

Naris-occluded TH-tdTom;CX_3_-GFP mice were anesthetized with isoflurane (4% induction; 1.5–2% maintenance) in O_2_ (1l/min) and received subcutaneous ketoprofen (5 mg/kg). Pinch withdrawal reflex was monitored throughout surgery. An ~1 mm square region over each OB was carefully thinned to 20–30 μm using a drill and microblade. This thinned skull preparation is minimally invasive and does not elicit microglial activation (Xu et al., 2007). A 0.1 g metal bar was attached to the skull for positioning. Mice were imaged with a Leica TCS SP5 microscope using a HCX APO 20x/1.00 NA water-immersion objective and Chameleon Vision II IR laser mode-locked at 910 nm. GFP emission was collected at 500–550 nm and tdTomato at >600 nm. Time-lapse z-stacks of the dorsal surface GL of open and occluded OBs were collected at 30 s intervals for 10 min. Voxel size: 0.09 (xy) ^*^ 0.99 (z) μm.

### Image analysis

Image analysis was performed in Fiji (Schindelin et al., 2012) unless otherwise noted. Brightness and contrast were linearly manipulated for display purposes only. Analysis of neuronal, microglial, and synaptic density was performed on widefield images of every fourth section through the entire OB. Quantification for these measures was restricted to appropriate regions of interest (ROIs). Neuronal density was quantified using the “Analyze Particles” command, which was facilitated through applying a median-filter of a predetermined radius and an ROI-determined threshold. Microglial density was analyzed in the same way, with TH-tdTom co-expression used to choose appropriate ROIs for different layers. TH-syptdTom mice express tdTomato-labeled synaptophysin in DA neurons. Synaptophysin, a widely used presynaptic marker, is localized to presynaptic terminals. Hence, fluorescence in the TH-syptdTom line is localized to the presynaptic terminals of DA neurons. As a result, the mean fluorescence intensity of the TH-syptdTom signal in the glomerular layer of raw images provided a surrogate measure of synaptic density.

Manual analysis of “contacting” and “wrapping” of neurons by microglia was performed on 3D renderings (generated using the Fiji “3D Viewer” plugin) of confocal z-stacks. Interactions were analyzed in the dorsal, medial, ventral, and lateral faces of each OB, in an anterior, central, and posterior coronal section. “Contacting” was most commonly observed in interactions between ramified microglia and DA neurons, but it was also observed in interactions between amoeboid microglia and DA neurons. The frequency of contacting was quantified as the percentage of tdTomato-labeled DA neuron somata that were clearly being touched by the terminal end of at least one GFP-labeled microglial process. “Wrapping” occurred when three criteria were met: (1) it occurred in a one-to-one fashion between a single microglia and a single DA neuron; (2) the microglial and neuronal somata were closely apposed; and (3) thick, primary microglial processes were oriented toward and wrapping around the DA neuron soma. We did not identify any instances in which both contacting and wrapping of a DA neuron occurred. Engulfment was detected by manual examination of confocal z-stacks as regions where tdTomato signal was surrounded by GFP signal, and confirmed by inspection of orthogonal views of that region.

Quantification of process morphology metrics and Sholl analysis were performed on 3D reconstructions of microglial processes that were generated from confocal z-stacks of microglia. For consistency, microglia from the lateral surfaces of the central coronal section of each OB were reconstructed. Reconstructions were generated semi-automatically using AutoPath to ensure consistency across reconstructions.

Analysis of microglial motility was carried out using *in vivo* 2-photon time-lapse images. z-stacks at each time point were collapsed into 2D maximum intensity projections to facilitate quantification of microglial perimeter, a median filter with a predetermined radius and an image-specific threshold were applied. ROIs containing individual microglia were generated automatically for perimeter measurements at each time point. Perimeter ROIs traced the outline of microglia in the 2D projection such that the perimeter measurement was comprised of individual processes and the interleaved portions of somatic perimeter. In instances of overlapping processes, corrections to ROIs were made manually.

### Statistics

Statistical analysis was performed in Prism (GraphPad). Data that were normally distributed with equal variance were analyzed using two-tailed *t*-tests, one-way ANOVA, and two-way repeated measures ANOVA. These data are presented as mean ± SD. Data that failed normality and/or equal variance tests were analyzed with Mann-Whitney tests, and are presented as median with interquartile range (IQR). α was set at 0.05 for all tests. In **Figures 2, 3B,C, 4, 6**, statistical analyses were performed on average values for individual control animals or individual OBs (naris-occluded mice). For **Figures 3D,E**, pairwise comparisons of summed values for individual OBs were compared. In **Figure 5**, statistical analyses were performed on individual cells. For these data, the standard deviation of percentage change in perimeter for cells within individual OBs (mean, 1.00%, range 0.66–1.32%) was similar to the standard deviation of mean values per OB (0.93 for occluded OBs, 0.91 for open OBs).

## Results

### Rapid activity-dependent decrease in DA neuron and synapse density

**Figure 1:**
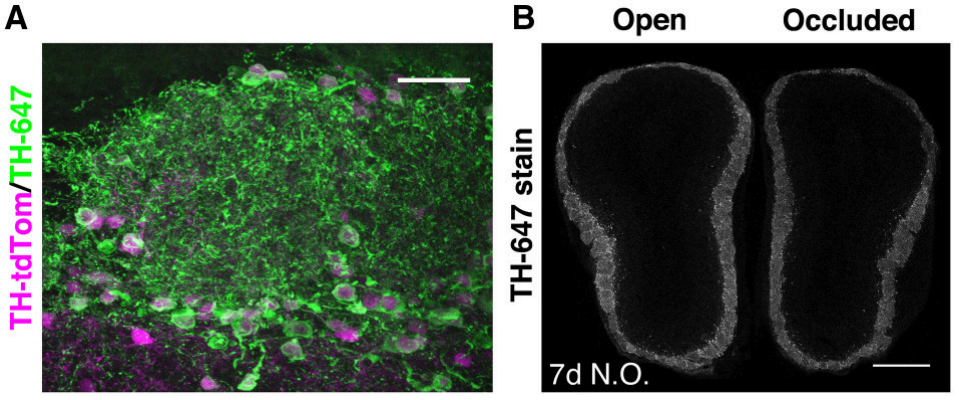
**Pronounced but ongoing loss of OB DA neurons after 7 days of naris occlusion**. **(A)** TH staining of the GL in a TH-tdTom mouse. Scale bar: 30 μm. **(B)** TH staining in a coronal section through both OBs of a naris-occluded mouse. Mean fluorescence intensity in the GL was 26.7% lower in the occluded than in the open OB. Scale bar: 500 μm.

To investigate whether microglia are involved in activity-dependent elimination of OB DA neurons, we employed a Cre-mediated expression strategy to irreversibly label DA neurons (Figure 1A, Methods). 78% of tdTomato-labeled neurons (*n* = 1150) in the GL expressed TH, comparable to previously reported values using similar genetic strategies (Adam and Mizrahi, 2011; Sawada et al., 2011). We also confirmed that our unilateral naris occlusion method resulted in a significant reduction in TH expression in the OB ipsilateral to the occluded naris (Figure 1B), as reported previously (Baker et al., 1993).

**Figure 2:**
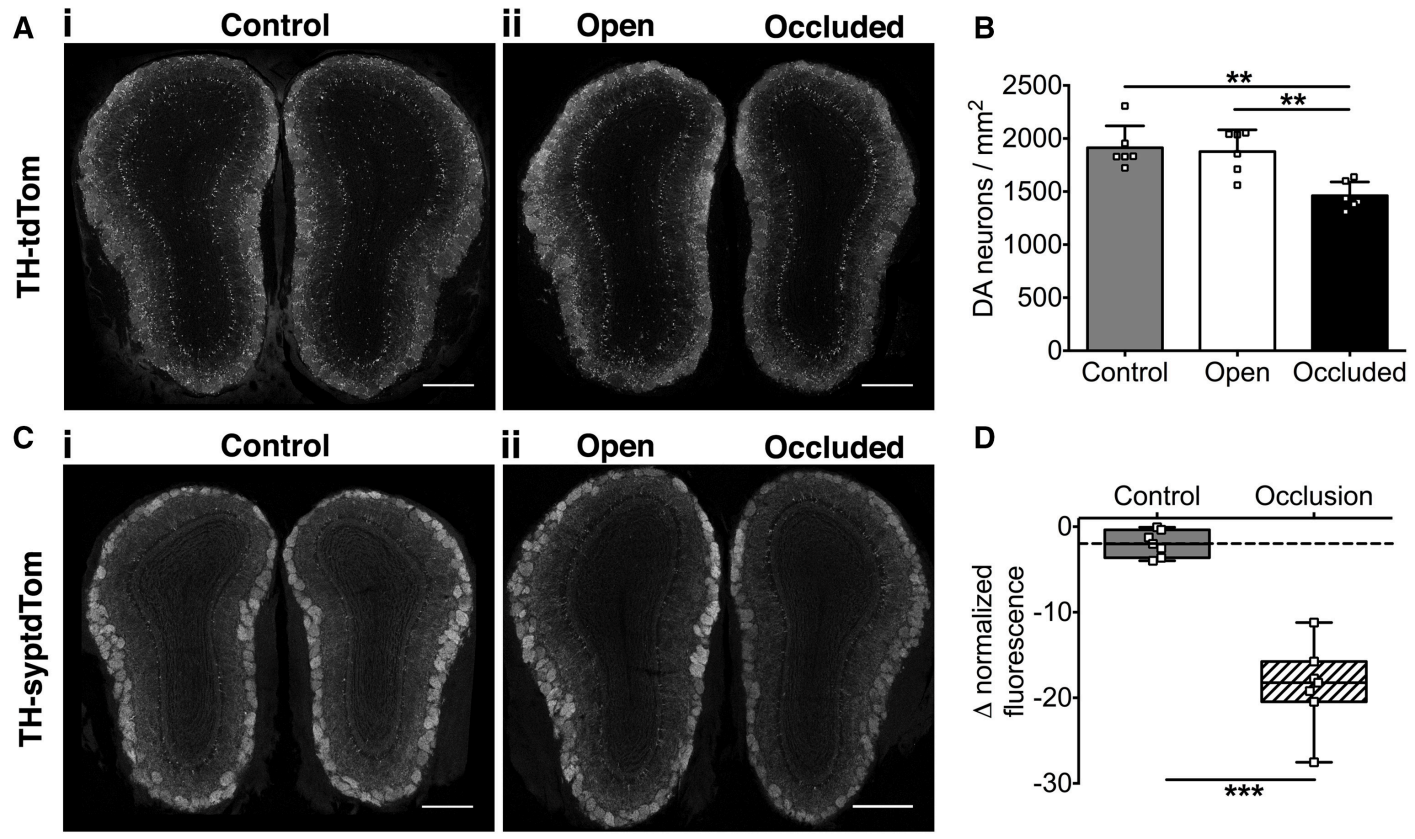
**Activity-dependent decrease in OB DA neuronal and synaptic density after 7 days of naris occlusion**. **(A)** DA neurons and **(C)** DA synapses in (i) control and (ii) naris-occluded mice. Scale bars: 500 μm. **(B)** Lower neuron density in occluded than open (^**^p = 0.004) or control (^**^p = 0.002) OBs; no difference between open and control OBs (*p* = 0.94) [one-way ANOVA with Tukey's test, *p* = 0.001, *F*_(2, 15)_ = 11.14; *n* = 6 pooled pairs of OBs from 6 control mice and 6 pairs of open and occluded OBs from 6 naris-occluded mice]. **(D)** Lower synapse fluorescence intensity in occluded OBs (^***^p < 0.001) [Mann-Whitney Test, *U* = 0; *n* = 7 pooled pairs of OBs from 7 control mice and 7 pairs of open and occluded OBs from 7 naris-occluded mice]. Box shows median and interquartile range; whiskers show minimum and maximum values.

We then assessed the effect of naris occlusion on DA neurons and their synapses in young adult mice. Using TH-tdTom mice, we found that just 7 days of naris occlusion resulted in a significant decrease in DA neuron density in the GL of the occluded OB relative to both the open OB (22.2% decrease) and unoccluded control mice (control OB, 23.6% decrease; Figures 2A,B), consistent with our pilot study (Materials and Methods). There was no difference in DA neuron density between control and open OBs (Figures 2A,B). Next, we assessed whether the density of synapses formed by DA neurons is also reduced after 7 days of naris occlusion. We quantified changes in DA presynaptic terminal density in TH-syptdTom mice by comparing GL syptdTom fluorescence between pairs of OBs in control and naris-occluded mice. syptdTom fluorescence was 18.6% lower in the occluded OB than the open OB, a significantly greater difference than could be accounted for by random variation between pairs of OBs in control mice (2.0%, Figures 2C,D). These data, together with a previous study showing ~40% loss of DA neurons after 28 days of naris occlusion (Sawada et al., 2011), suggest that 7 days was an optimal time point at which to assess the role of microglia in activity-dependent DA neuron loss.

### Rapid increase in microglial density in the olfactory bulb

**Figure 3:**
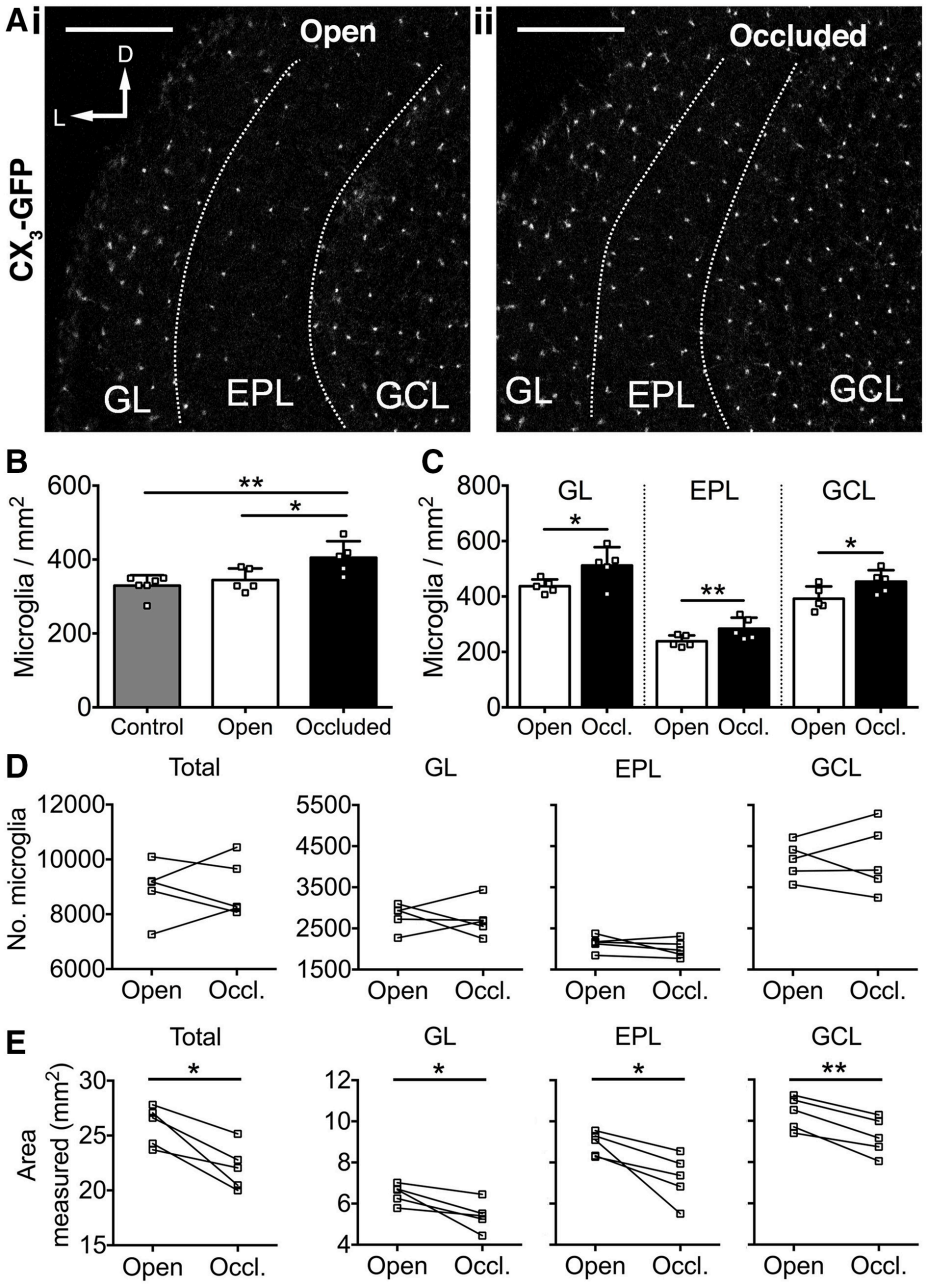
**Activity-dependent increase in microglial density after 7 days of naris occlusion**. **(A)** Microglia in (i) open and (ii) occluded OBs. Scale bars: 200 μm. **(B)** Higher microglial density in occluded OB than open (^*^p = 0.042) or control (^**^p = 0.009) OBs; no difference between open and control OBs (*p* = 0.76) [one-way ANOVA with Tukey's test; *p* = 0.009, *F*_(2, 13)_ = 6.914]. **(C)** Higher microglial density in glomerular (GL; ^*^p = 0.048, *t* = 2.83), external plexiform (EPL; ^**^p = 0.009, *t* = 4.78), and granule cell (GCL; ^*^p = 0.011, *t* = 4.53; paired *t*-tests) layers of occluded OBs. B,C: *n* = 6 pooled pairs of OBs from 6 control mice and 5 pairs of open and occluded OBs from 5 naris-occluded mice. **(D)** No difference in number of microglia between open and occluded OBs in total (*p* = 0.97, *t* = 0.03) or for the GL (*p* = 0.79, *t* = 0.28), EPL (*p* = 0.28, *t* = 1.24), or GCL (*p* = 0.91, *t* = 0.11; paired *t*-tests). **(E)** Decreased total OB area (^*^p = 0.011, ^*^p = 0.030, ^*^p = 0.027, ^**^p = 0.003, *t* = 4.54), GL area (*p* = 0.030, *t* = 3.31), EPL area (*p* = 0.027, *t* = 3.39) and GCL area (*p* = 0.003, *t* = 6.59; paired *t*-tests) in occluded vs. open OBs. **(D,E)**: *n* = 5 pairs of open and occluded OBs from 5 naris-occluded mice.

We next investigated whether 7 days of naris occlusion affected microglial density in the OB, using CX_3_CR1-GFP transgenic mice (Jung et al., 2000) to specifically label microglia. We found that 7 days of naris occlusion significantly increased microglial density in the occluded OB, relative to both the open (17.5% increase) and control (22.9% increase) OBs (Figures 3A,B), with no difference between the open and control OBs (Figure 3B). Furthermore, microglial density in the occluded OB was significantly higher across the glomerular (+17.2%), external plexiform (+19.0%), and granule cell (+25.6%) layers (Figure 3C). Further pairwise analysis of open vs. occluded OBs showed that there was no difference in the absolute number of microglia in the GL, EPL, or GCL (Figure 3D). However, there was a significant decrease in the area of the GL, EPL, and GCL (Figure 3E), suggesting that the increase in microglial density in the occluded OB was due to a reduction in OB volume rather than an increase in the number of microglia. Together, this increase in microglial density and concurrent decreases in the density of DA neurons and synapses suggest that microglia may regulate DA neuron number and connectivity. Because there was no difference in either microglial or DA neuron density between open and control OBs (Figures 2C, 3B), we restricted further analyses to comparisons between open and occluded OBs.

### Loss of olfactory activity drives changes in microglia morphology

**Figure 4:**
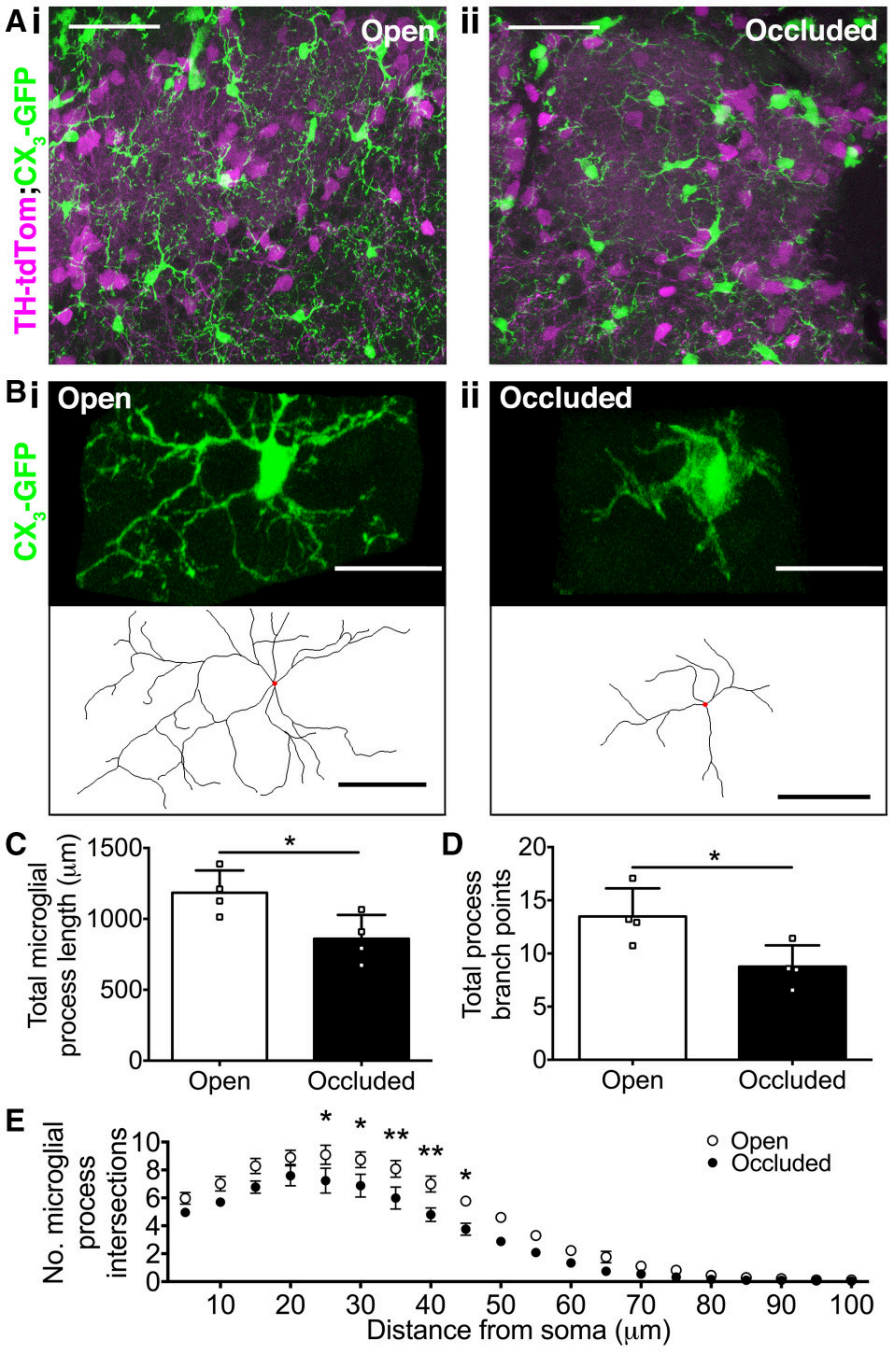
**Loss of olfactory input drives changes in microglial morphology**. **(A,B)** (i) More ramified microglial morphology in open OB vs. (ii) more amoeboid microglial morphology in occluded OB. **(A)** Scale bars: 50 μm. **(B)** Lower panels show reconstructions (red dots: somata). Scale bars: 20 μm. **(C)** Shorter process length in occluded OBs (^*^p = 0.031, *t* = 2.82, *t*-test). **(D)** Fewer process branch points in occluded OBs (^*^p = 0.029, *t* = 2.85, *t*-test). **(E)** Lower process complexity in occluded OBs [effect of occlusion, *p* < 0.001, *F*_(1, 120)_ = 72.31; ^*^p < 0.05; ^**^p < 0.01 at particular radii from soma; two-way repeated measures ANOVA with Sidak's test]. **(C–E)**: *n* = 4 pairs of open and occluded OBs from 4 naris-occluded mice. All comparisons are of mean values per OB. A total of 54 microglia in occluded OBs and 52 microglia in open OBs were reconstructed (7–18 microglia per OB).

Microglial morphology exists along a broad spectrum, ranging from highly ramified “resting” microglia that survey their surroundings, to amoeboid “activated” microglia that exhibit phagocytic behavior (Kreutzberg, 1996; Hanisch and Kettenmann, 2007). Therefore, we next asked whether microglial morphology is affected by naris occlusion, and found clear differences between open and occluded OB microglia (Figures 4A,B). To quantify these changes, we reconstructed microglia from the GL of the OB in naris occluded TH-tdTom;CX_3_-GFP mice. After 7 days of naris occlusion, there was a 27.3% decrease in microglial process length in the occluded OB (Figure 4C) and a 35.0% decrease in the number of process branch points per microglia (Figure 4D). In contrast, there was no significant difference in the number of primary processes extending from microglial somata between the open OB [median (IQR): 4 (3–5)] and the occluded OB [median (IQR): 4 (3–4.25)] (Mann-Whitney test, *p* = 0.14, *U* = 1178). Therefore, the observed changes in microglial process length and number of branch points are not a consequence of fewer primary microglial processes.

To further quantify the effect of naris occlusion on microglial process morphology, we performed 3D Sholl analysis of reconstructed microglia. There were significantly fewer Sholl intersections 25–45 μm from the soma for microglia in the occluded OB, relative to those in the open OB (Figure 4E), and the critical value (maximum number of process intersections with a Sholl radius) was 18.2% smaller in the occluded (7.9 ± 0.8) than in the open (9.6 ± 0.6) OB (*t*-test, *p* = 0.13, *t* = 1.73). Furthermore, the critical radius (distance from the soma at which the critical value occurs) was 11.5% closer to the soma in the occluded (19.8 ± 1.5 μm) than in the open (23.8 ± 2.5 μm) OB (*t*-test, *p* = 0.21, *t* = 1.39), consistent with microglia in the occluded OB having less complex process morphology. Together, these data demonstrate that loss of sensory input drives microglia toward more amoeboid morphologies indicative of activation. Activated microglia in the occluded OB may interact differently with their local environment, shifting from “surveillance” to “engulfment” (Hanisch and Kettenmann, 2007).

### *In vivo* imaging reveals greater microglial dynamics in the occluded OB

**Figure 5:**
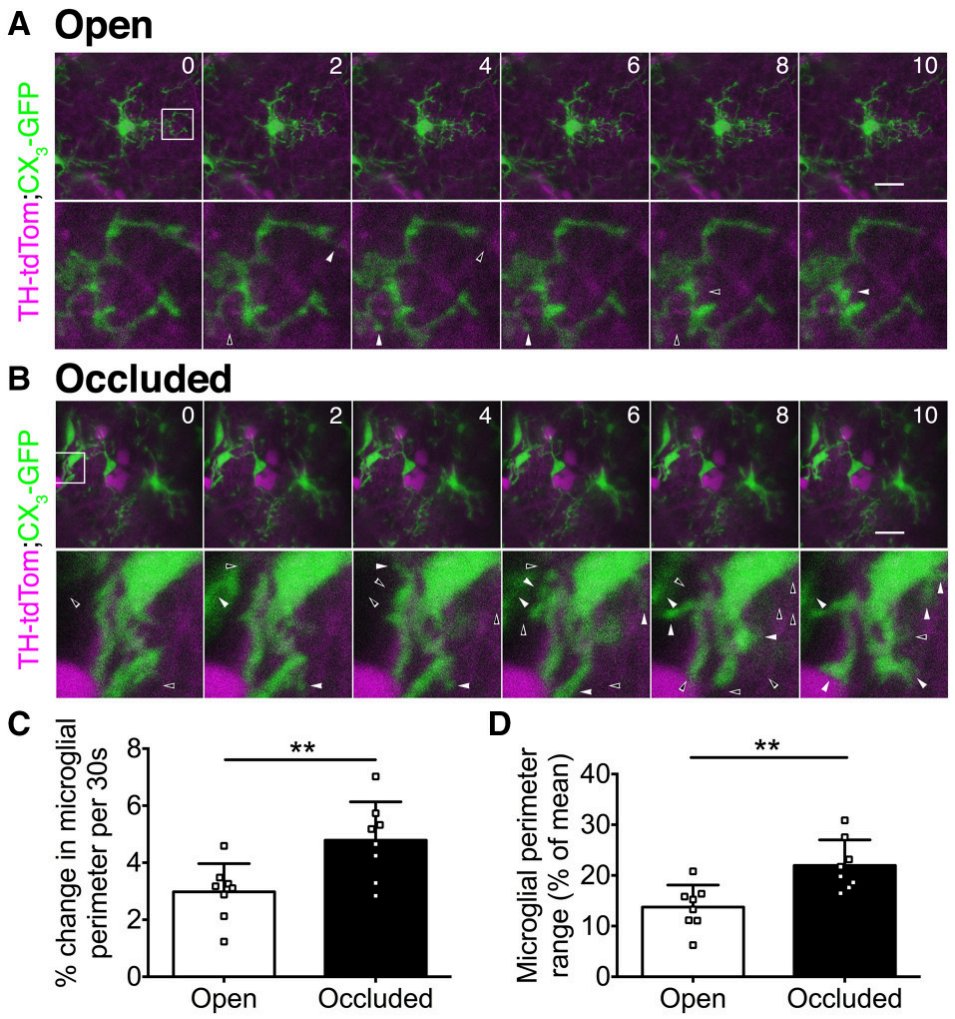
*****In vivo*** 2-photon imaging reveals activity-dependent changes in microglial dynamics**. **(A,B)** Upper: time-lapse image series showing microglial dynamics at select time points. Numbers: time (minutes). Images were captured at 30 s intervals. Scale bars: 20 μm. Lower: zoomed images of boxed regions. Left-to-right: solid-open arrowhead pairs indicate process retraction; open-solid arrowhead pairs indicate process extension. **(C)** Greater perimeter change in occluded OBs (^**^p = 0.008, *t* = 3.06, *t*-test). **(D)** Greater perimeter range over time in occluded OBs (^**^p = 0.004, *t* = 3.49, *t*-test). **(C,D)**: *n* = 8 microglia from 2 open OBs and 8 microglia from 2 occluded OBs from 2 naris-occluded mice.

Microglia rapidly extend and retract their processes (Nimmerjahn et al., 2005), and this property depends on neuronal activity levels in visual cortex (Wake et al., 2009; Tremblay et al., 2010). To determine whether microglial process dynamics in the OB are also activity dependent, we used *in vivo* 2-photon imaging to compare microglia in the open and occluded OBs of TH-tdTom;CX_3_GFP mice after 7 days of naris occlusion (Supplementary Video 1). While microglial process dynamics were evident in both the open (Figure 5A) and occluded (Figure 5B) OBs, we observed much greater process extension and retraction in the occluded than in the open OB (Figures 5A,B). We quantified these dynamics by comparing microglial perimeter over the imaging period. The average rate of change of microglial perimeter was 60.5% greater (Figure 5C), and microglial perimeter range was 59.8% higher (Figure 5D), in the occluded than in the open OB. These data demonstrate that microglia in the occluded OB restructure their processes more rapidly and with greater range than in the open OB.

### Activity-dependent shift in microglial-neuronal interactions

**Figure 6:**
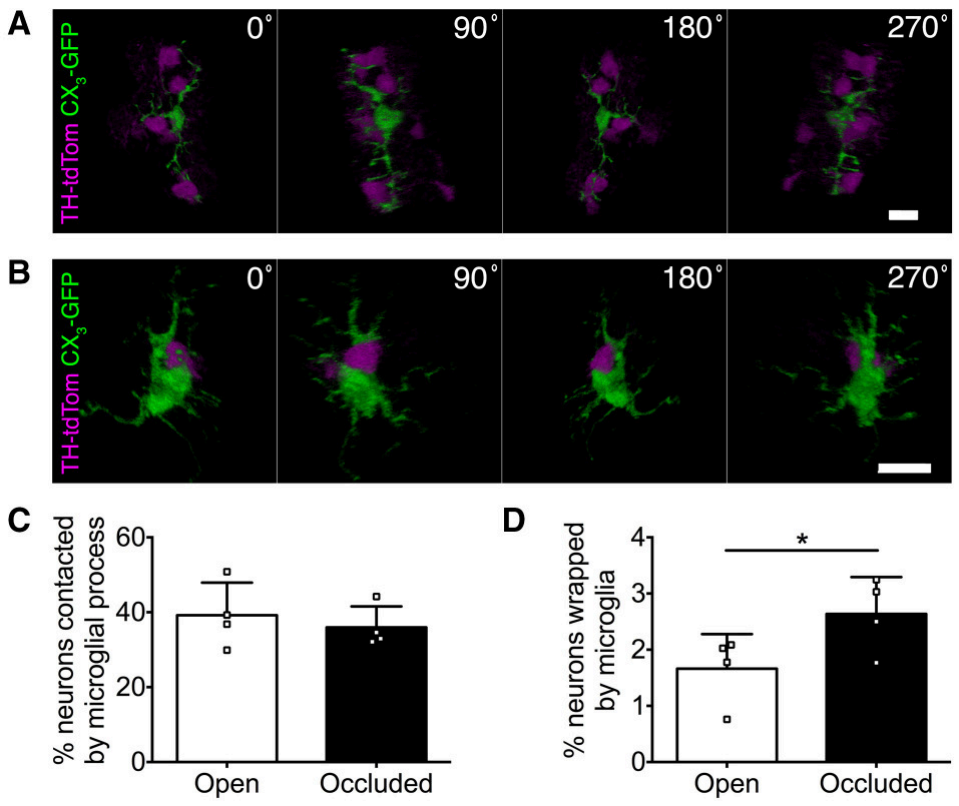
**Microglial-neuronal interactions are activity-dependent**. **(A,B)** 3D reconstructions showing microglia **(A)** contacting multiple DA neurons or **(B)** wrapping a DA neuron. **(C)** Similar percentage of DA neurons contacted by microglia in occluded and open OBs (*p* = 0.22, *t* = 1.56, paired *t*-test). **(D)** Greater microglial wrapping of DA neurons in occluded OBs (^*^p = 0.017, *t* = 4.78, paired *t*-test). **(C,D)**: *n* = 4 pooled pairs of control OBs from 4 control mice and 4 pairs of open and occluded OBs from 4 naris-occluded mice.

Microglia can make direct physical contact with neurons (Nimmerjahn et al., 2005; Wake et al., 2009; Tremblay et al., 2010), so we next compared microglia-DA neuron interactions in the open and occluded OBs of TH-tdTom;CX_3_-GFP mice. From initial observations, we parsed out two classes of physical interactions, which we termed “contacting” and “wrapping.” Contacting was defined as microglial processes touching neuronal somata (Figure 6A, Supplementary Video 2). Wrapping occurred in a one-to-one fashion defined by contact between microglial and neuronal somata and short, thick microglial processes wrapping around the neuronal soma (Figure 6B, Supplementary Video 3). Interestingly, the rate of microglial contacting of neuronal somata was unaffected by naris occlusion (Figure 6C). In contrast, the rate of wrapping increased in the occluded OB (Figure 6D), consistent with our previous observation of more activated microglial morphology. Taken together, our data suggest that changes in olfactory input to the OB drive altered microglial-neuronal interactions.

### Microglia engulf components of olfactory DA neurons in the occluded OB

**Figure 7:**
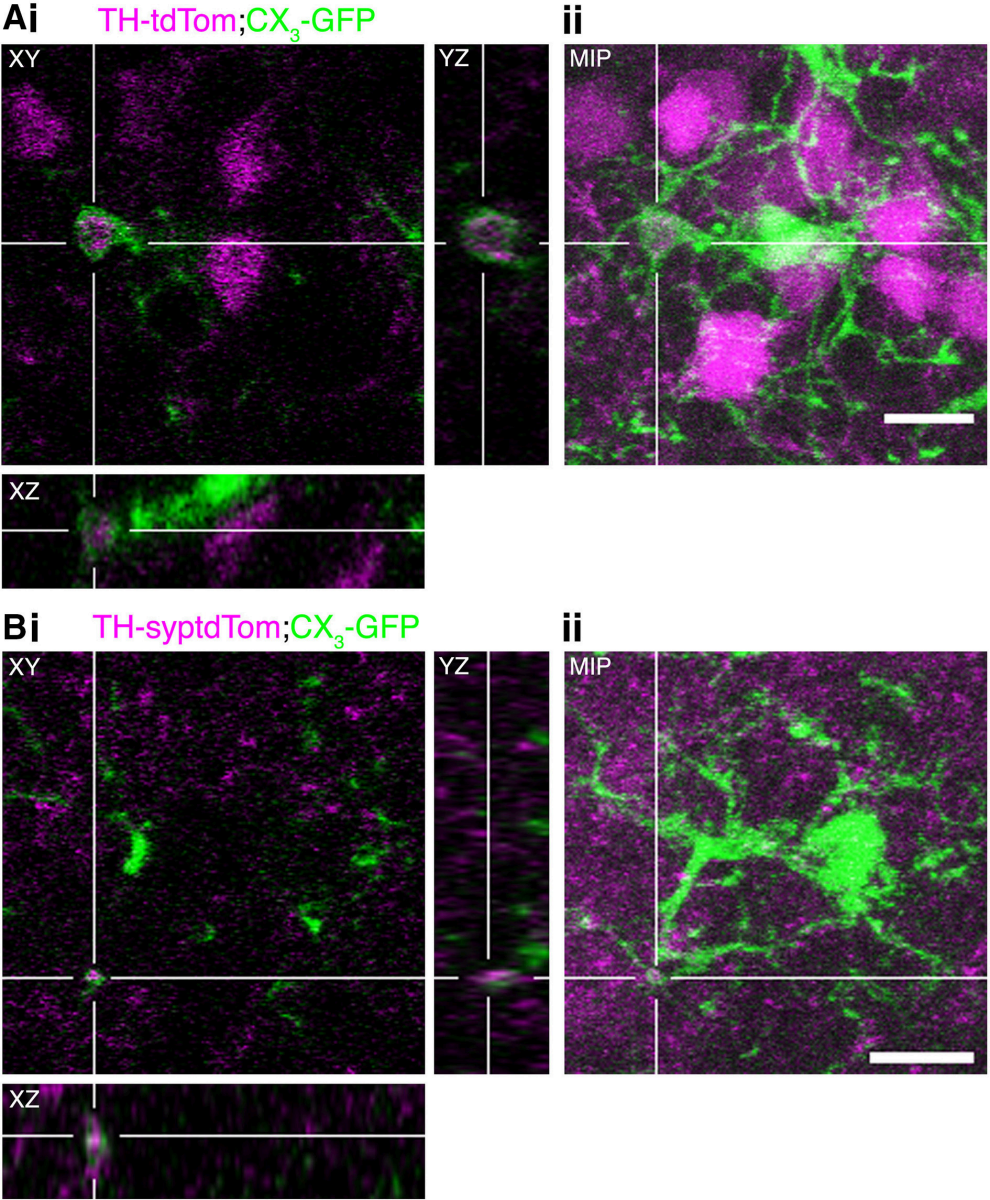
**Microglia engulf components of DA neurons in the occluded OB**. **(A,B)** Examples of microglial engulfment of tdTom-labeled material in the occluded OB of **(A)** a TH-tdTom;CX_3_-GFP mouse and **(B)** a TH-syptdTom;CX_3_-GFP mouse. (i) 3D section views. (ii) Maximum intensity projection. All scale bars: 10 μm.

In addition to phagocytosing the remains of apoptotic neurons, microglia can actively regulate neurons by phagocytosing synapses (Schafer et al., 2012) and even entire viable neurons in culture (Neher et al., 2011) and in zebrafish embryos (Peri and Nüsslein-Volhard, 2008). Through analysis of confocal image stacks from TH-tdTom;CX_3_-GFP and TH-syptdTom;CX_3_-GFP mice we identified multiple compelling examples of microglial engulfment of DA neuron components (e.g., Figure 7A) and synapses (e.g., Figure 7B) in occluded OBs. In conjunction with the increase in microglial density and changes in microglial-neuronal interactions, the presence of tdTom-labeled material within microglia in occluded OBs suggests that microglia play an important role in the activity-dependent loss of DA neurons.

## Discussion

### Rapid initial loss of OB DA neurons and their synapses following naris occlusion

To investigate a potential role for microglia in the loss of OB DA neurons caused by naris occlusion (Sawada et al., 2011), we first needed to identify a time point at which DA neuron loss was significant, but still ongoing. Surprisingly, after just 7 days of naris occlusion, there was already a 22% decrease in DA neuron density, with 30 and 38% decreases seen after 14 and 21 days, respectively. This implies that DA neuron loss occurs rapidly within the first week of naris occlusion, then proceeds more gradually to reach the maximal 40% loss seen after 4 weeks (Sawada et al., 2011). Interestingly, compensatory mechanisms such as the continuing addition of new DA neurons during naris occlusion (Sawada et al., 2011) or increased recruitment of DA neurons such as that seen following 6-hydroxydopamine lesion of the GL (Lazarini et al., 2014) are insufficient to maintain DA neuron density. Alternatively, DA neuron numbers may be specifically reduced to meet the current needs of the OB network during naris occlusion. Consistent with this, the 19% decrease in DA neuron synapse density after 7 days of naris occlusion shows that synaptogenesis by remaining DA neurons was not up-regulated to offset neuronal loss.

### Activity-dependent increase in activated microglia in the OB

As DA neurons and synapses underwent a dramatic loss following naris occlusion, microglial density increased significantly across all OB layers. This finding is consistent with the increased microglial density throughout the OB following deafferentation (Lazarini et al., 2012). Similarly, microglial density increased in the cochlear nucleus following cochlear ablation (Fuentes-Santamaría et al., 2012). In contrast, an early study found no change in microglial density in the OB following neonatal naris occlusion (Caggiano and Brunjes, 1993). Thus, it is possible that microglia respond differently to loss of sensory input in the developing and adult OB. Alternatively, given that microglial density is elevated by developmental cell death (Ashwell, 1989), the high levels of cell death in the GL during early postnatal life (Fiske and Brunjes, 2001) may have saturated microglial density. The increase in microglial density in the occluded OB that we report resulted from a decrease in OB area rather than an increase in the absolute number of microglia. Microglia tile the brain, enabling them to continuously survey surrounding tissue (Nimmerjahn et al., 2005). Hence, the increase in microglial density in the occluded OB means that each microglia in the occluded OB is responsible for surveying a smaller volume than in the open OB.

Microglia also adopted morphologies consistent with activation in the occluded OB; this change was discernible by eye and was corroborated through quantitative analysis of microglial processes. Such morphological changes are consistent with reports of increased microglial activation in the OB following both naris occlusion and chemical ablation of the OE (Fiske and Brunjes, 2000; Chang et al., 2003), and are again mirrored in the cochlear nucleus following cochlear ablation (Fuentes-Santamaría et al., 2012). Activated microglia are known to exhibit phagocytic behavior (Hanisch and Kettenmann, 2007), consistent with the idea that microglia engulf DA synapses and perhaps even entire DA neurons following naris occlusion.

### Increased microglial process dynamics in the occluded OB

This study presents the first report of *in vivo* two-photon imaging of microglia in the OB. Mirroring previous studies in somatosensory cortex (S1) and V1 (Nimmerjahn et al., 2005; Wake et al., 2009; Tremblay et al., 2010), OB microglia have highly dynamic processes that rearrange on a timescale of minutes and directly contact neighboring neurons. However, unlike in V1, where sensory deprivation decreased microglial process motility and contact with neurons (Wake et al., 2009; Tremblay et al., 2010), or in S1, where increased neuronal activity due to pharmacological blockade of inhibition increased process dynamics (Nimmerjahn et al., 2005), in the OB, sensory deprivation significantly increased microglial dynamics. This discrepancy could be due to functional differences between the OB and sensory cortices, which result in different microglial behaviors following activity manipulations. Alternatively, the more dramatic plasticity seen in the OB, which includes substantial neuronal loss, may invoke different or additional microglial functions to those seen in sensory cortex, where plasticity is limited to removal of synapses.

### Activity-dependent changes in microglial-neuronal interactions

The role of microglia as active effectors in sculpting neural circuits vs. passive garbage collectors remains debated. We found activity-dependent changes in microglia-DA neuron interactions, reflected by a significant increase in “wrapping” of DA neurons by microglia in the occluded OB. We also found clear evidence for microglial engulfment of components of DA neurons in occluded OBs. What role then, do microglia play in the activity-dependent loss of DA neurons and their synapses? One possibility is that microglia play an active role in eliminating DA neurons. Close apposition of microglia with neuronal somata is also seen in the cochlear nucleus following cochlear ablation (Fuentes-Santamaría et al., 2012) and is thought to be a precursor to engulfment (Barcia et al., 2012). Alternatively, microglia may have a neuroprotective function, as shown recently for neurons in the adult motor cortex where microglial wrapping of neuronal somata displaced inhibitory synapses, increasing neuronal activity and activating a neuroprotective intracellular signaling cascade (Chen et al., 2014). Notably, this “synaptic stripping” does not involve phagocytosis (Chen et al., 2014). Finally, microglia may simply act as “garbage collectors,” phagocytosing dead or dying DA neurons and cellular debris, with cell death occurring by a distinct, microglia-independent mechanism. These three mechanisms are not mutually exclusive, and could operate in parallel, or to different extents at different time points following loss of sensory input. Furthermore, the mechanisms underlying loss of mature DA neurons vs. lack of survival and integration of newborn DA neurons, both of which could contribute to the net loss of DA neurons, could be distinct.

Understanding how microglia target neurons and synapses for removal will be important in elucidating the expanding roles ascribed to glia during activity-dependent plasticity. That microglia have also been implicated in the loss of substantia nigra DA neurons in Parkinson's disease models (Gao et al., 2002; Ouchi et al., 2005) further underscores their potential importance in modulating neural function. Thus, determining the basis for selective neuronal susceptibility to microglial modification could provide a new pathway toward understanding neurological disorders.

## Author contributions

BG, LB, and CC designed experiments; BG, CC performed experiments, analyzed data and prepared the figures; BG, LB, and CC wrote the manuscript. All authors approved the final version of the manuscript.

## Funding

This work was supported by the NINDS Intramural Program (1-ZIA-NS003002) and a Human Frontier Science Program Long-Term Fellowship to CC.

### Conflict of interest statement

The authors declare that the research was conducted in the absence of any commercial or financial relationships that could be construed as a potential conflict of interest. The reviewer JM and handling Editor declared their shared affiliation, and the handling Editor states that the process nevertheless met the standards of a fair and objective review.
