# Supplementary material for: Olfactory Sensory Activity Modulates Microglial-Neuronal Interactions during Dopaminergic Cell Loss in the Olfactory Bulb
Source: Front Cell Neurosci. 2016 Jul 15;10:178. doi: 10.3389/fncel.2016.00178 (PMC4945633; doi:10.3389/fncel.2016.00178)
Supplement: Supplementary file 4 [file DataSheet1.DOCX]

Supplementary Material

**Olfactory sensory activity modulates microglial-neuronal interactions during dopaminergic cell loss in the olfactory bulb**

Bryce D. Grier, Leonardo Belluscio*, Claire E J Cheetham*

*** Correspondence:** Claire Cheetham or Leonardo Belluscio
[cheetham@andrew.cmu.edu](mailto:cheetham@andrew.cmu.edu) or [belluscl@ninds.nih.gov](mailto:belluscl@ninds.nih.gov)

**Supplementary Videos**

**Supplementary Video 1. Rapid microglial dynamics in the OB in vivo.**

Time-lapse images of a single microglia in the occluded OB *in vivo*. Images were acquired at 30s intervals for 10min. Maximum soma diameter: 9.5 μm.

**Supplementary Video 2. Contacting of DA neurons by a microglia.**

3D render of a confocal z-stack showing a microglia (green) contacting DA neurons (red) in the occluded OB. Scale bar: 20 μm.

**Supplementary Video 3. Microglial wrapping of a DA neuron.**

3D render of a confocal z-stack showing wrapping of a DA neuron (red) by a microglia (green) in the occluded OB. Scale bar: 20 μm.

**
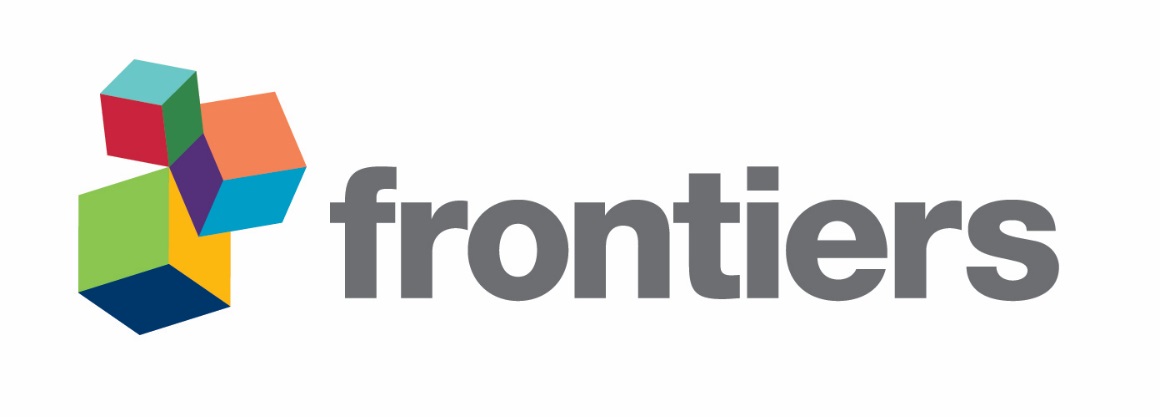
**
